# Edible Bird Nest Protects the Kidney From Gentamicin Induced Acute Tubular Necrosis

**DOI:** 10.3389/fphar.2021.726005

**Published:** 2021-09-29

**Authors:** Christopher T S. Lim, Norhafizah M., D. Sani, S. N. Tan, C. W. Lim, Brian P. Kirby, A. Ideris, J. Stanslas

**Affiliations:** ^1^ Department of Medicine, Faculty of Medicine and Health Sciences, UPM Serdang, Serdang, Malaysia; ^2^ Department of Pathology, Faculty of Medicine and Health Sciences, UPM Serdang, Serdang, Malaysia; ^3^ School of Pharmacy and Biomolecular Sciences, RCSI University of Medicine and Health Sciences, Dublin, Ireland; ^4^ Department of Veterinary Clinical Studies, Faculty of Veterinary Medicine, UPM Serdang, Serdang, Malaysia

**Keywords:** edible bird nest, renal protective, sialic acid, epidermal growth factor, medicine

## Abstract

Every year, there are about 13.3 million cases of acute kidney injury (AKI). Although AKI is a preventable and treatable disease, if left untreated, it has high risk of multiple organ failure and progression to end stage kidney disease. Acute tubular necrosis (ATN) has been recognised as one of the major causes of AKI. Till to date, there is no effective supplement or medication in treating or reversing AKI. Most of the treatment strategies involve preventative measure to minimise the occurrence of AKI or to reverse the cause of AKI. Hence one of the primary area of research interests is to explore the potential treatment for AKI. Edible bird nests (EBN) are edible food produce by the swiftlet’s saliva, which is rich in sialic acids. Sialic acids are monosaccharides that play a vital role in maintaining the integrity and proper function of the human organs, including kidneys. EBN also contains epidermal growth factor, which is widely believed to have rejuvenation and tissue repairing properties. We initiate this study to study the potential reno-protective effect of edible bird’s nests by studying the Wistar rat model of gentamicin-induced AKI. Besides renal profiles, renal histology was also semiquantitatively assessed. In our study, pre-treatment with EBN prevented and ameliorated the gentamicin-induced AKI. To a lesser extent, post-treatment with EBN also protected the kidney from the toxic effect of gentamicin. Our findings are highly indicative that EBN possesses reno-protective properties.

## Introduction

Based on Kidney disease Improving Global Outcome (KDIGO) guidelines, AKI can be defined by either increase in serum creatinine by > 0.3mg/dl, increase in serum creatinine by > 1.5 times baseline, or a reduction in urine volume <0.5 ml/kg/h for 6 h (Khwaja, 2012). It can be further subdivided into Acute Kidney Injury Network (AKIN) Stages based on changes in serum creatinine level (Bagshaw et al., 2008). AKI, regardless of its stages, carries significant morbidity and mortality risks.

The causes of AKI can be divided into three broad categories: prerenal (caused by decreased blood perfusion to the kidneys), intrinsic renal (caused by a process within the glomeruli and tubules), and postrenal (caused by disruption of drainage of urine). Intrinsic renal causes are also relevant sources of AKI with acute tubular necrosis (ATN) as the most common type of AKI encounter in the patients that are hospitalised. The cause is usually an ischaemic insult or post exposure to nephrotoxic agent.

Edible bird nest (EBN), produced from the saliva of the swiftlets, is consumed worldwide and is often regarded as a medicinal food (Marcone, 2005). EBN comprises many exceedingly nutritious substances like glycoproteins, carbohydrates, minerals and both essential and non-essential amino acids. It is also abundant in hormones, vitamins and fatty acids. According to our previous works, the highest composition contained inside the EBN is protein. Carbohydrates were the next highest ingredient found in the EBN. There is also evidence that there is the presence of sialic acid and epidermal growth factors in the EBN (Tan et al., 2020). Other growth factors contained inside EBN, such as vascular endothelial growth factor (VEGF) and cytokines such as interleukin-6 (IL-6), play a vital role in cellular communication. As intercellular mediators, these molecules play an essential role in regulating survival, growth, differentiation, and functions of cells (Roh et al., 2012).

Although consumption of EBN is believed to have beneficial effect to the kidney for many centuries, there are no previous trials that study its effect on preservation of the kidney function. The beneficial effect is believed to be mediated through down regulation of the inflammatory pathway, reparation of the renal tissues via the presence of epidermal growth factor and maintenance of tissue integrity via sialic acid.

Therefore, this present study was initiated to evaluate the reno-protective effect of EBN using a Wister rat model of gentamicin-induced acute tubular necrosis (ATN).

## Materials and Methods

Raw cleaned EBN samples were sourced from a swiftlet ranch located on a piece of agriculture land (2°52′51.4″N 103°16′33.1″E) in Pahang, Malaysia. The raw, cleaned EBN was then immerse in reverse osmosis water for 1 hour until it turned soft. Impurities from the nests were then carefully removed by using tweezers under the aid of a magnifying glass. After the dirt was removed, the EBN was then left to dry under the fan in room temperature. Cleaned nests were then sent to a local accredited lab for proximate analysis, toxicology and bacteriology analyses to ensure the quality of the product are met as per the established standard (Department of Standards of Malaysia, 2011). The cleaned EBN was then cooked according to the Chinese technique of double-boiled cooking method for 30 min until it has a gelatinous texture.

### Renal Injury Model

This study aims to evaluate the renoprotective effect of using EBN in pre-treatment and post-treatment phase of GTN induced ATN model ATN will be induced in rats by gentamicin. The method used is as described by Moreira (Moreira et al., 2014). For the pre-treatment phase, the rats will be fed EBN (at various doses) orally 7 days before the induction of ATN via injection of gentamicin. In contrast, for the post-treatment arm, rats will be fed EBN (at various doses) immediately after treatment with gentamicin. The control groups for both experiments are rats with tubular injuries but without the pre or post-treatment with EBN. The studied parameters in our study were renal profiles, serum osmolarity and renal histopathology. Renal histopathological slides were read, in a blinded manner, by an experienced histopathologist who would then record the renal pathological changes of ATN as according to Endothelial, Glomerular, Tubular, Interstitial (ETGI) scoring system (Khalid et al., 2016). Blood was collected before and after treatment for measurement of the renal profiles.

### Animal Handling and Housing

We used ninety-six female, 12–14 weeks old Wistar rats in this study. The rats were sourced from Takrif Bistari Enterprise, Selangor, Malaysia. They weighed in the 250–300 g range. All of the rats were held in groups in plastic cages for 10 days for them to adapt to the research laboratory setting. The research laboratory has a preset ambient temperature of 25 ± 2°C with alternating 12 h dark and light cycle. The rats were nourished with a constant pellet formula rodent diet recommended for rats (Specialty Feeds, Pte Limited, Western Australia). In contrast, the water supply was provided at an *ad libitum* basis. Our animal research was carried out in conformity with the standards of laboratory animal care as dictated by our local institution Animal Care Use Committee (approval number UPM/IACUC/AUP-R064/2014).

### Experimental Animal

Ninety-six Wistar rats were randomly separated into six groups, with each group containing eight rats.

A total of forty-eight rats were used in the six groups of pre-treatment arm: Group I: normal control; Group II: Gentamicin - injected rats without pre-treatment with EBN as negative control; Group III-V: Gentamicin - injected rats which have been pre-treated with different doses of EBN (125, 250 and 500 mg/kg respectively); Group VI: Gentamicin -injected rats which have been pre-treated with N-acetylcysteine as the positive control.

A total of forty-eight rats were used in the six groups of post-treatment arm: Group I: normal control; Group II: Gentamicin-injected rats without EBN given as post-treatment to act negative control; Group III-V: Gentamicin - injected rats which were post-treated with different doses of EBNs (125, 250 and 500 mg/kg respectively); Group VI: Gentamicin -injected rats subsequently receiving N-acetylcysteine (60 mg/kg) as the positive control group.

### EBN and Gentamicin Treatment

EBNs were diluted accordingly to achieve the desired dose concentration 125, 250 and 500 mg/kg correspondingly. The various doses were designed to be equivalent to a full, half and quarter dose oral consumption of EBN by human (5 g of cleaned EBN for a 60 kg human body weight). EBNs at different doses is fed orally to the rats (groups III to V) for 7 days before the administration of the intraperitoneal (IP) injection at a concentration of 100 mg/kg Gentamicin (GTN) (Garasent, Duopharma (M) Sdn Bhd, Malaysia). The GTN is dissolved in the normal saline. N-acetylcysteine was used at positive control and fed orally to the rats in group 6 before the IP GTN injection. For post-treatment phase, IP injection of 100 mg/kg GTN (Garasent, Duopharma (M) Sdn Bhd, Malaysia) was administered to the rats before EBNs at a graded does of 125, 250, and 500 mg/kg were fed orally to the rats for a further 7 days. Similarly, N-acetylcysteine was used as a positive control, and this was given orally to the group 6 rats post-GTN injection. The dose of the vehicle is 10 ml/Kg.

### Sample Collection

At the end of the research, all the rats were sacrificed via the administration of carbon dioxide gas. The abdomens were carefully dissected, and the kidneys were harvested. The kidneys were processed using routine histological techniques. The renal tissues were then stained with hematoxylin and eosin. The blood samples are taken via cardiac puncture with 2.5 ml of specimen collected in fluoride blood tubes. The blood was analysed using Olympus © Chemistry Analysers using the ISE modules. Samples are stored at Pharmacotherapeutics Laboratory, Faculty of Medicine and Health Sciences, Universiti Putra Malaysia for posterity purpose.

### Statistical Analysis

Results in our study were analysed using SPSS version 25 and expressed as mean ± standard deviation (SD). The data generated were analysed with Anova one-way analysis of variance while post hoc analysis was done using Bonferroni method. A p-value less than 0.05 is considered statistically significant in our study.

## Results

### Renal Histopathology

**FIGURE 1:**
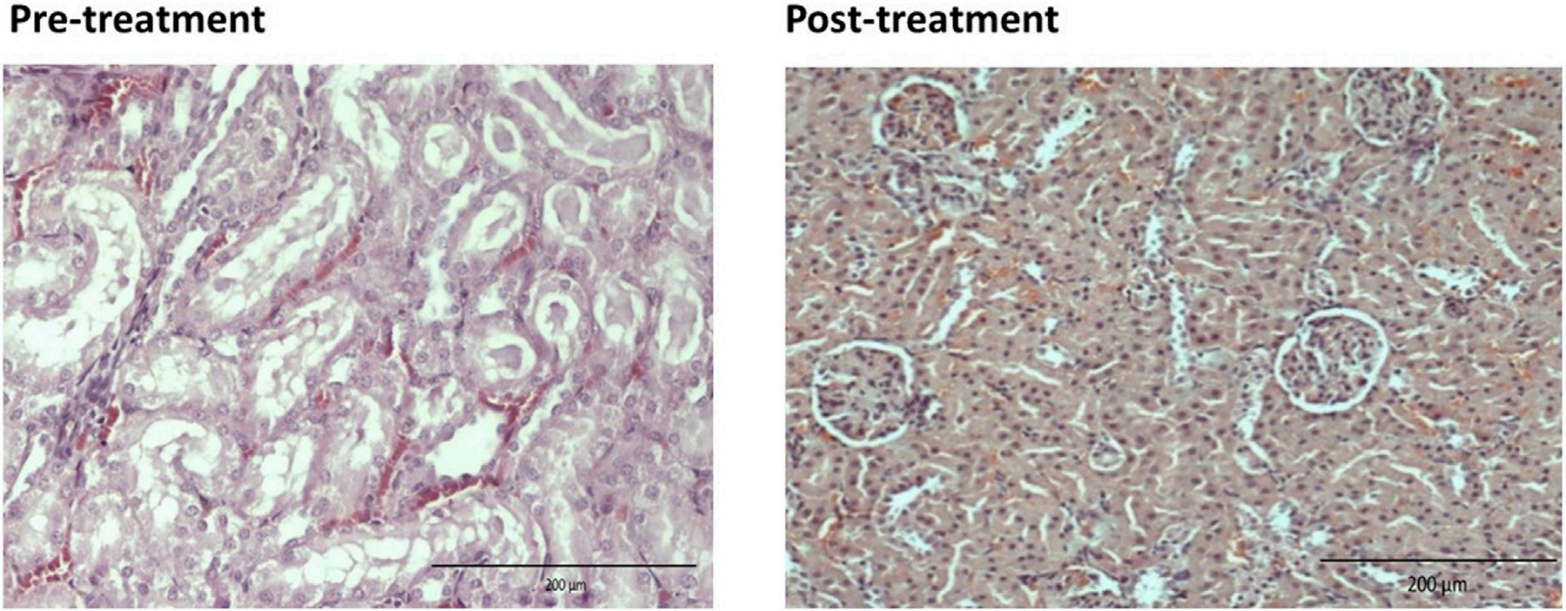
Control-Normal renal tubles and glomeruli (Hematoxylin and Eostin stain).

**FIGURE 2:**
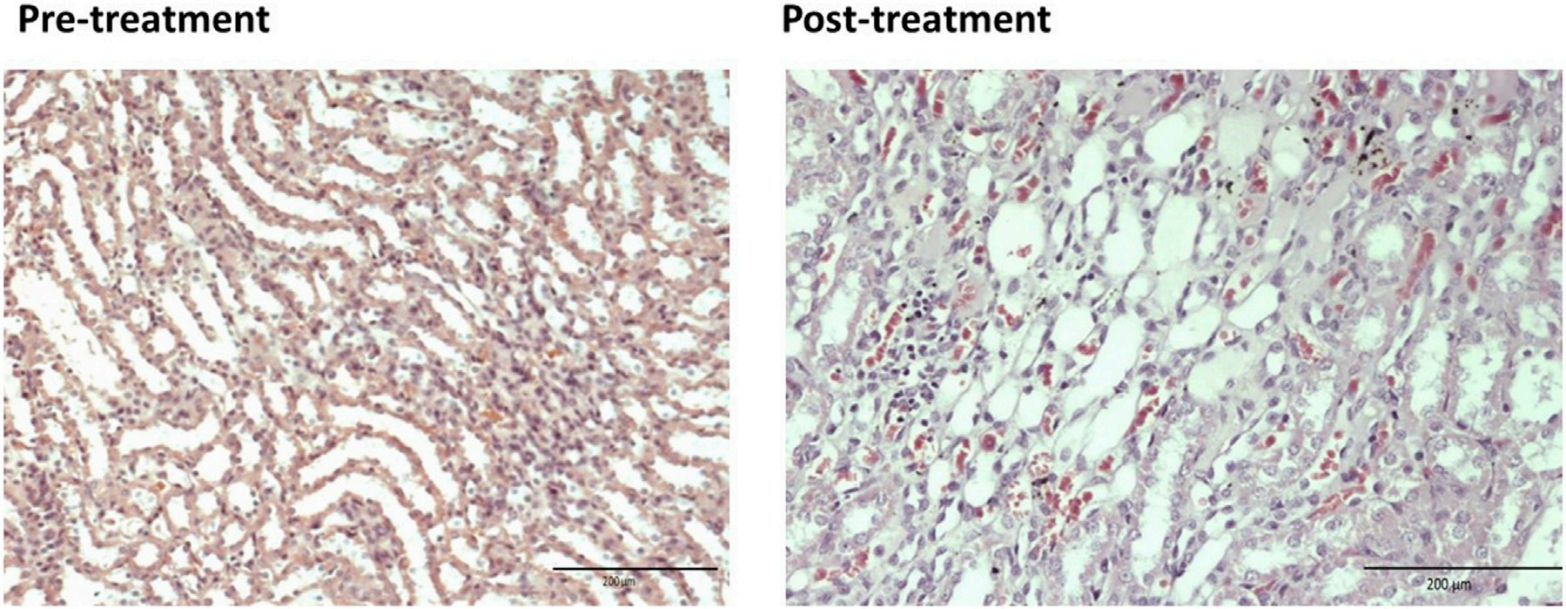
Negative contol (GTN and saline) Severe ATN changes in the tubles.

**FIGURE 3:**
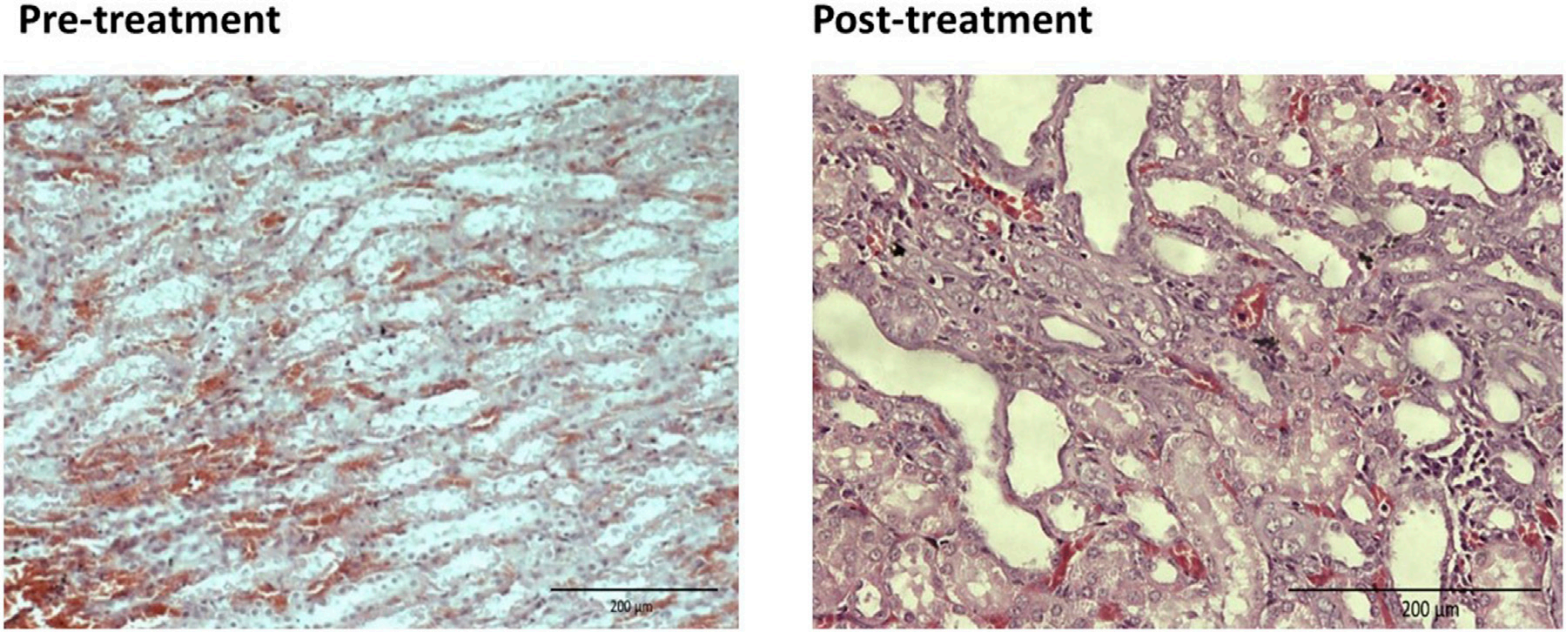
Postive control (GTN and NAC) Severe ATN changes.

The control kidneys which were treated with normal saline showed normal histopathological findings (Figure 1). The negative control kidneys, which were given gentamicin and normal saline, showed the classical picture of severe ATN damages, which is the hallmark feature of intrinsic AKI damages. These changes include markedly dilated renal tubules with flattening of the epithelium, diffuse denudation of the renal tubules, tubular atrophy and intratubular cast formation (Figure 2). Similar severe ATN features were found in the positive control kidneys (Figure 3).

**FIGURE 4:**
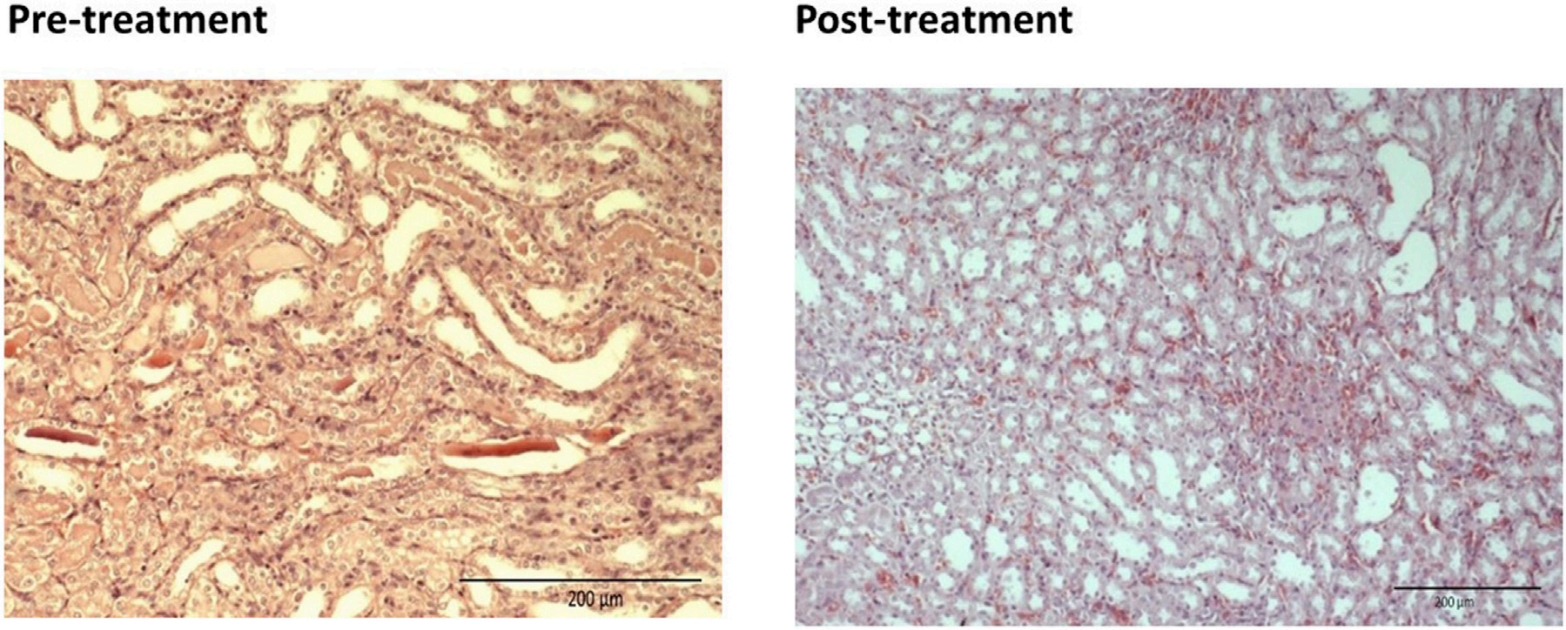
Pre-treatment with EBN 125mg/kg and GTN-relatively normal renal tubles.

**FIGURE 5:**
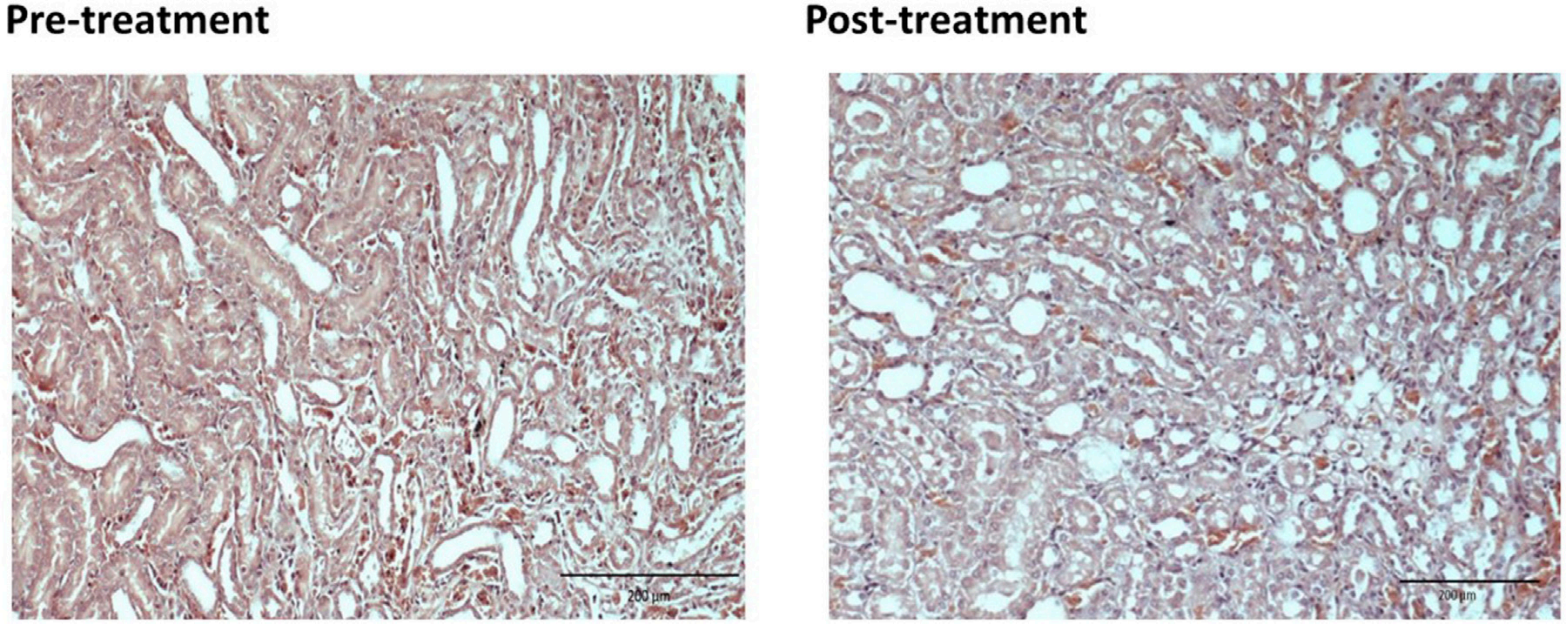
Pre-treatment with EBN 250mg/kg and GTN-mild ATN.

**FIGURE 6:**
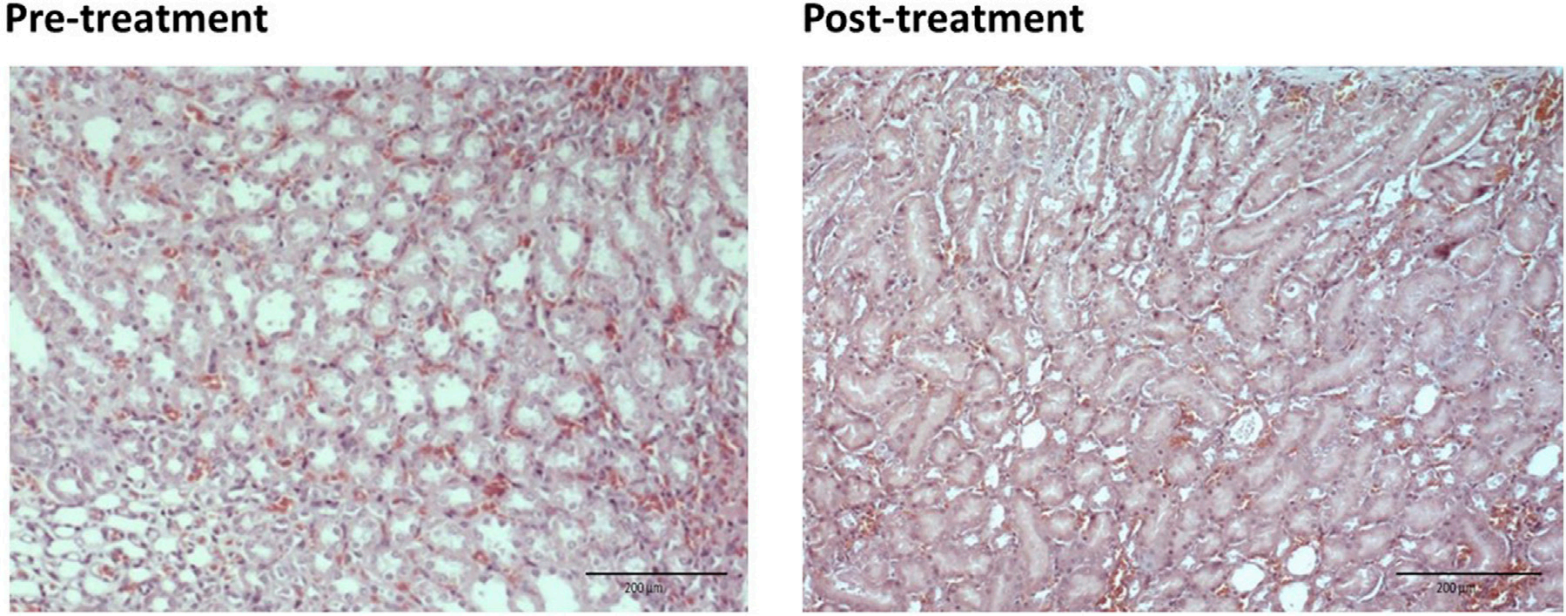
Pre-treatment with EBN 500mg/kg and GTN-mild ATN.

For the pre-treated group with different dose of EBNs, the 125 mg/kg group has near-normal renal tubules with no discernible ATN changes (Figure 4). The pre-treated group, which received 250 mg/kg and 500 mg/kg of EBNs, has mild ATN changes (Figures 5, 6). As for post-treated group, all different doses of EBN (125, 250, and 500 mg/kg) have resulted in moderate ATN changes (Figures 4–6).

The changes were scored using the EGTI system, as shown in Tables 1, 2 (Khalid et al., 2016). A higher score denotes worse renal histopathological damage. Pre-treatment with 125, 250, and 500 mg/kg have signifcantly lesser scores than GTN group (*p* < 0.05). The post-treatment groups 125, 250, and 500 mg/kg scores were lesser than GTN group but higher than pre-treatment groups.

**TABLE 1:** The EFTI scoring system for ATN.

Group	Tubular	Endothelial	Glomerular	Tubulo/Interstitial	P value
CONTROL					
Normal Control	0	0	0	0	
GTN	3+	0	0	2+	
PRE GROUP					
EBN 125 + GTN*	1+	0	0	0	0.025
EBN 250 + GTN*	1+	0	0	1+	0.038
EBN 500 + GTN*	1+	0	0	1+	0.038
NAC + GTN	3+	0	0	1+	NA
POST GROUP					
EBN 125 + GTN	2+	0	0	1+	0.205
EBN 250 + GTN	2+	0	0	1+	0.205
EBN 500 + GTN	2+	0	0	1+	0.205
NAC + GTN	3+	0	0	1+	NA

EFTI: Endothelial, Glomerular, Tubular, Interstitial (ETGI) scoring system.

GTN, Gentamicin; NAC, N-acetylcysteine.

A bivariate analysis were done to determine the significant different of Pre and Post EBN concentration with GTN (*p < 0.05).

**TABLE 2:** Plots of EGTI scores.

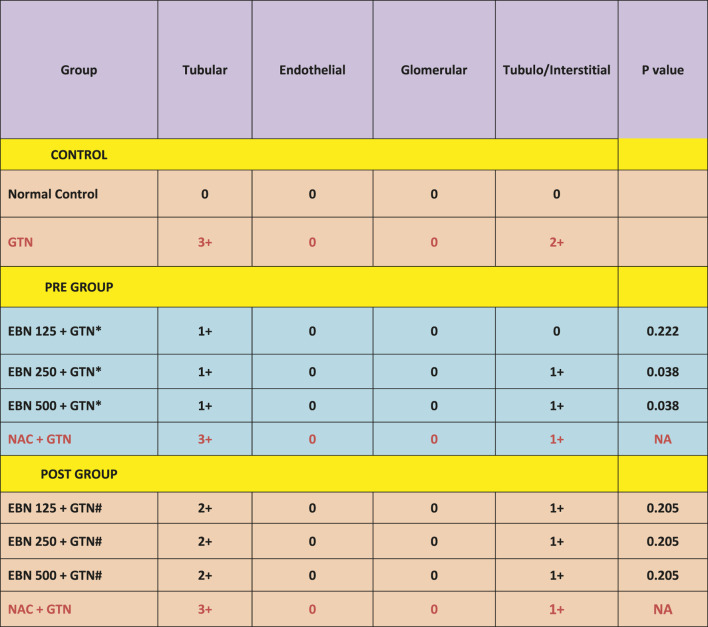
EGTI : Endothelial, Glomerular, Tubular, Interstitial (EGTI) scoring system GTN : Gentamicin, NAC : N-acetylcysteine A bivariate analysis were done to determine the significant different of Pre and Post EBN concentration with GTN (p < 0.05) *#compare mean analysis
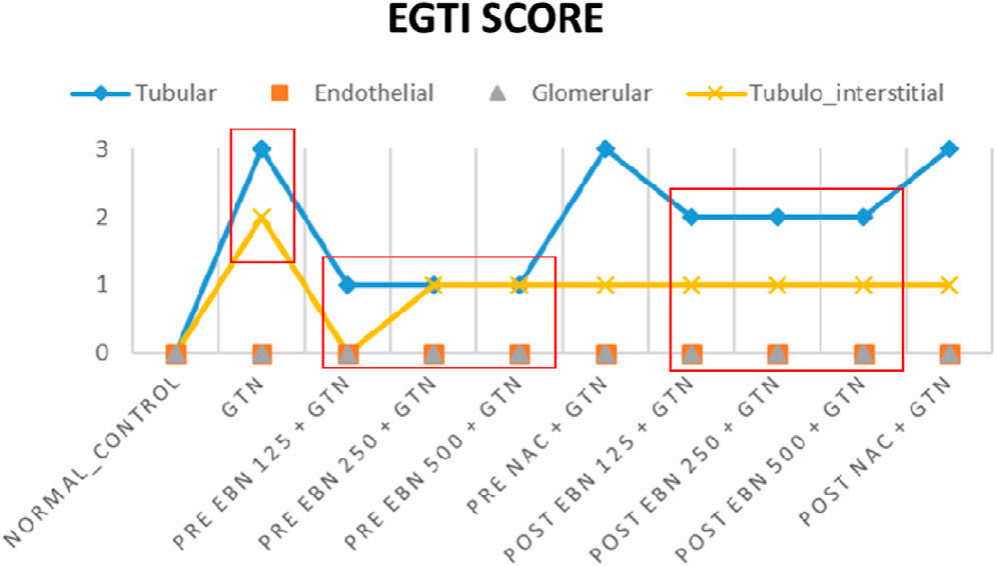

Line graph showing the EGTI score. There is significant lower EGTI scoring achieved in the groups received pre-treatment as compared to positive and negative controls treated with GTN

### Serum Renal Profiles

#### Pre-Treatment With EBN

**FIGURE 7:**
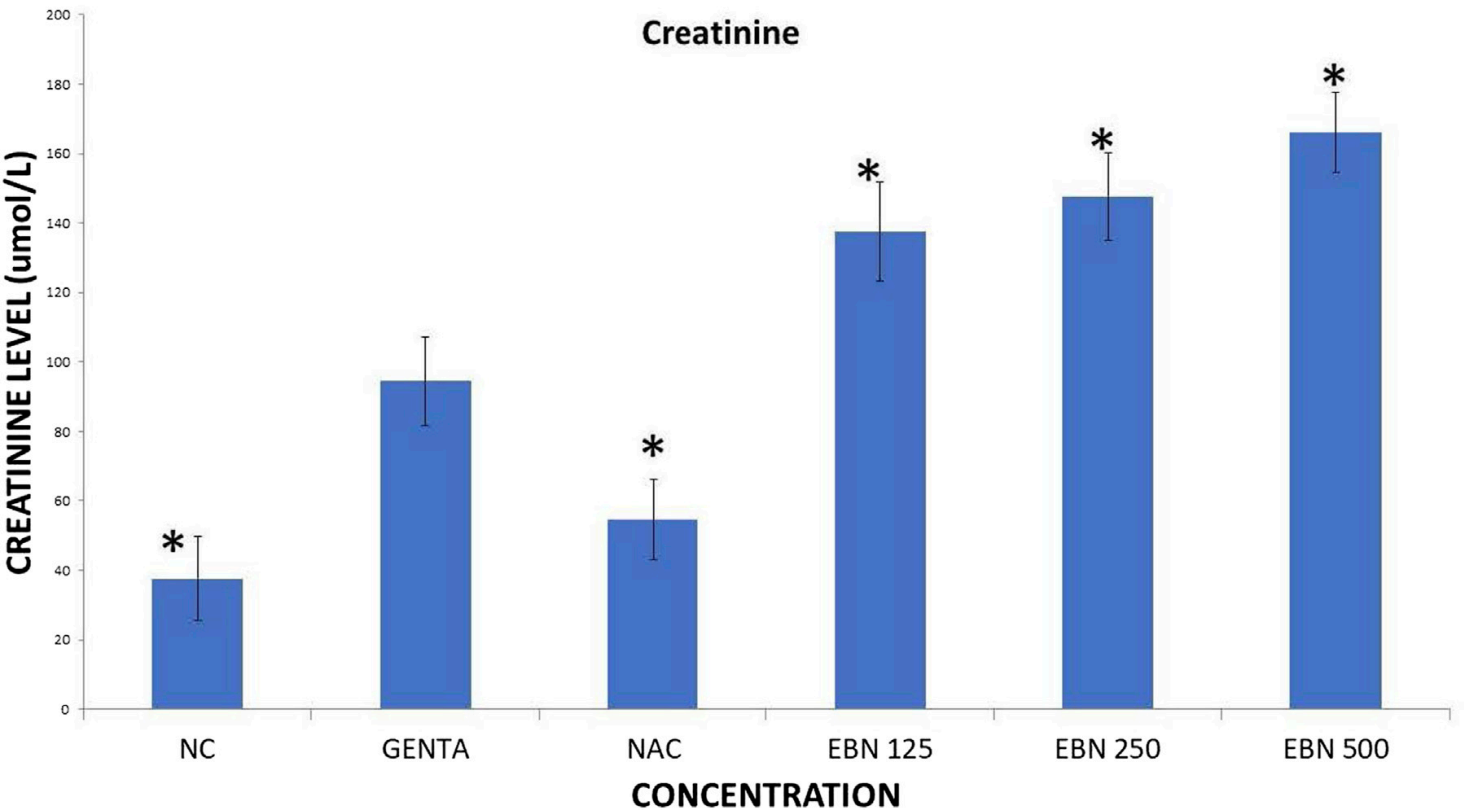
Changes of serum creatinine chnages vs. different pre-treatment group (*p < 0.05 compare to gentamicin group).

**FIGURE 8:**
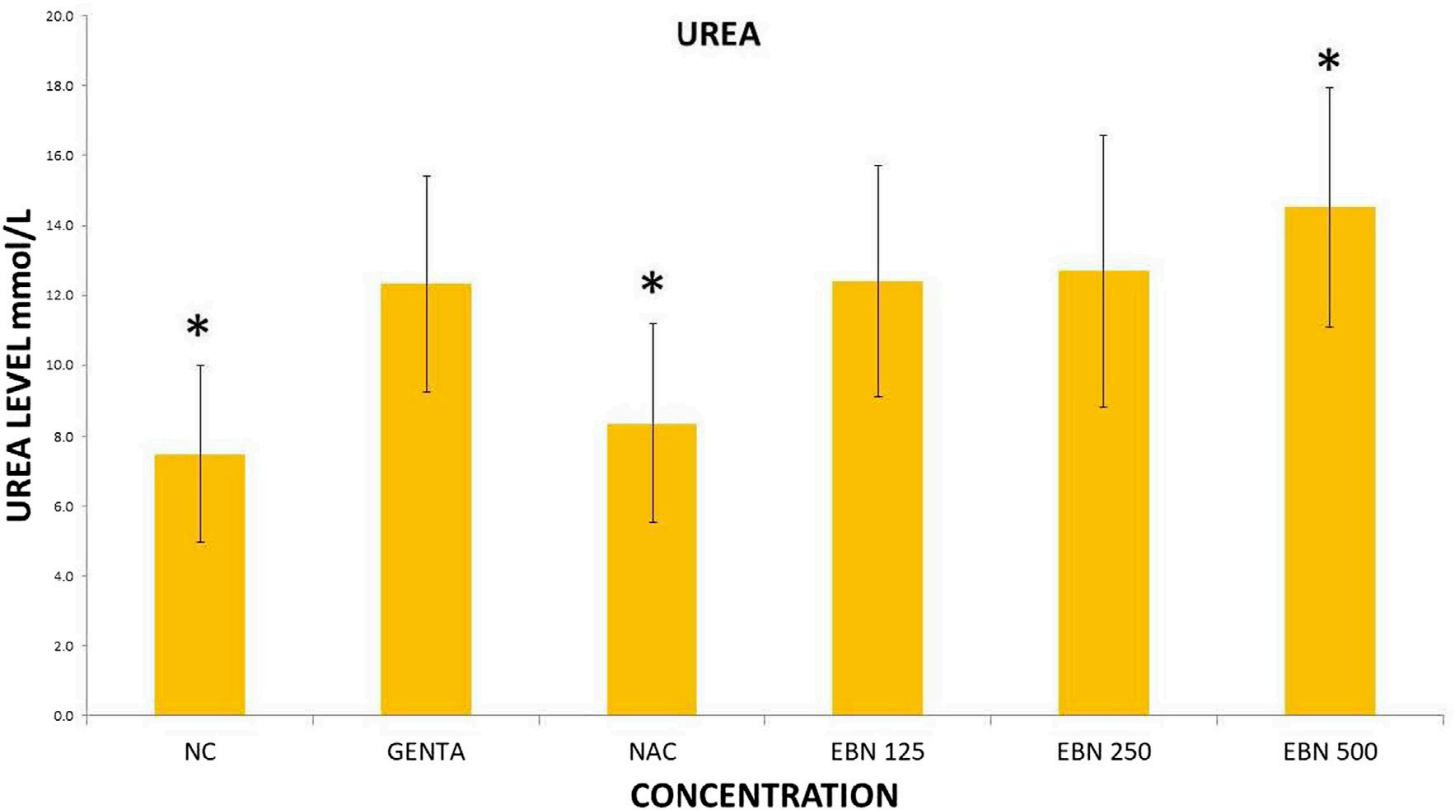
Changes of serum urea changes vs. different pre-treatment group (*p < 0.05 compare to gentamicin group).

**FIGURE 9:**
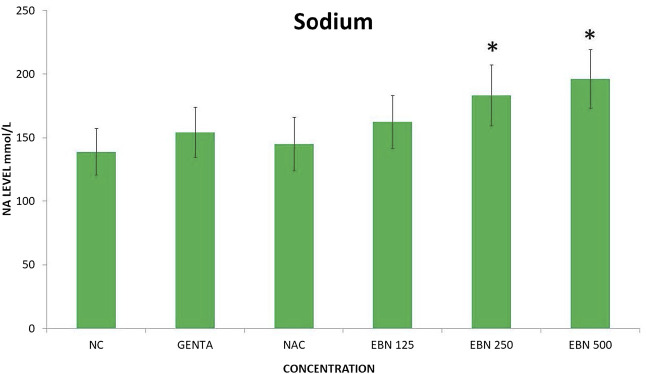
Changes of serum sodium vs. different pre-treatment group (*p < 0.05 compare to gentamicin group).

**FIGURE 10:**
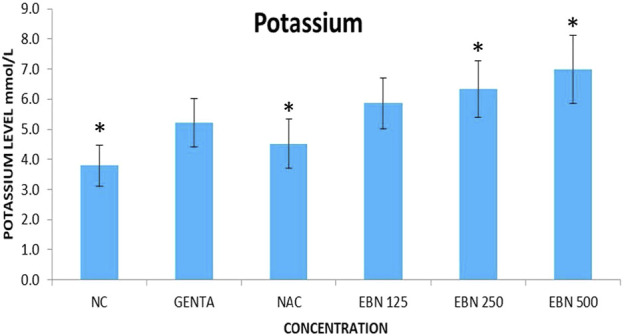
Changes of serum potassium vs. different pre-treatment group (*p < 0.05 compare to gentamicin group).

Serum renal profiles were taken for both pre and post-treatment groups. Serum creatinine was significantly higher in the pre-treated 125, 250, and 500 mg/kg groups as compared to the GTN group. Serum urea was only significantly higher in the pre-treated 500 mg/kg group as compare to GTN cohort. Serum sodium and potassium were higher in the pre-treated group, which received 250 and 500 mg/kg of EBNs (Figures 7–10).

#### Post Treatment With EBN

**FIGURE 11:**
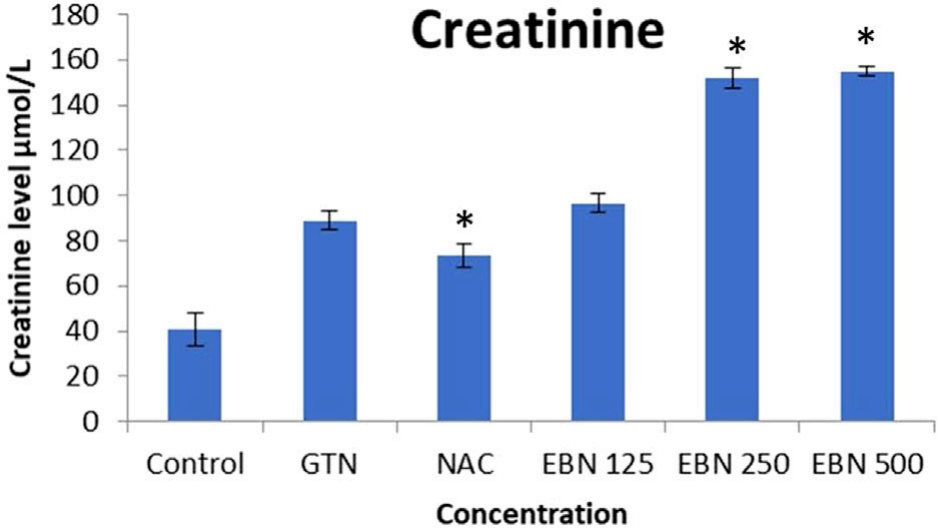
Changes of serum creatinine vs different post-treatment group (*p < 0.05 compare to gentamicin group).

**FIGURE 12:**
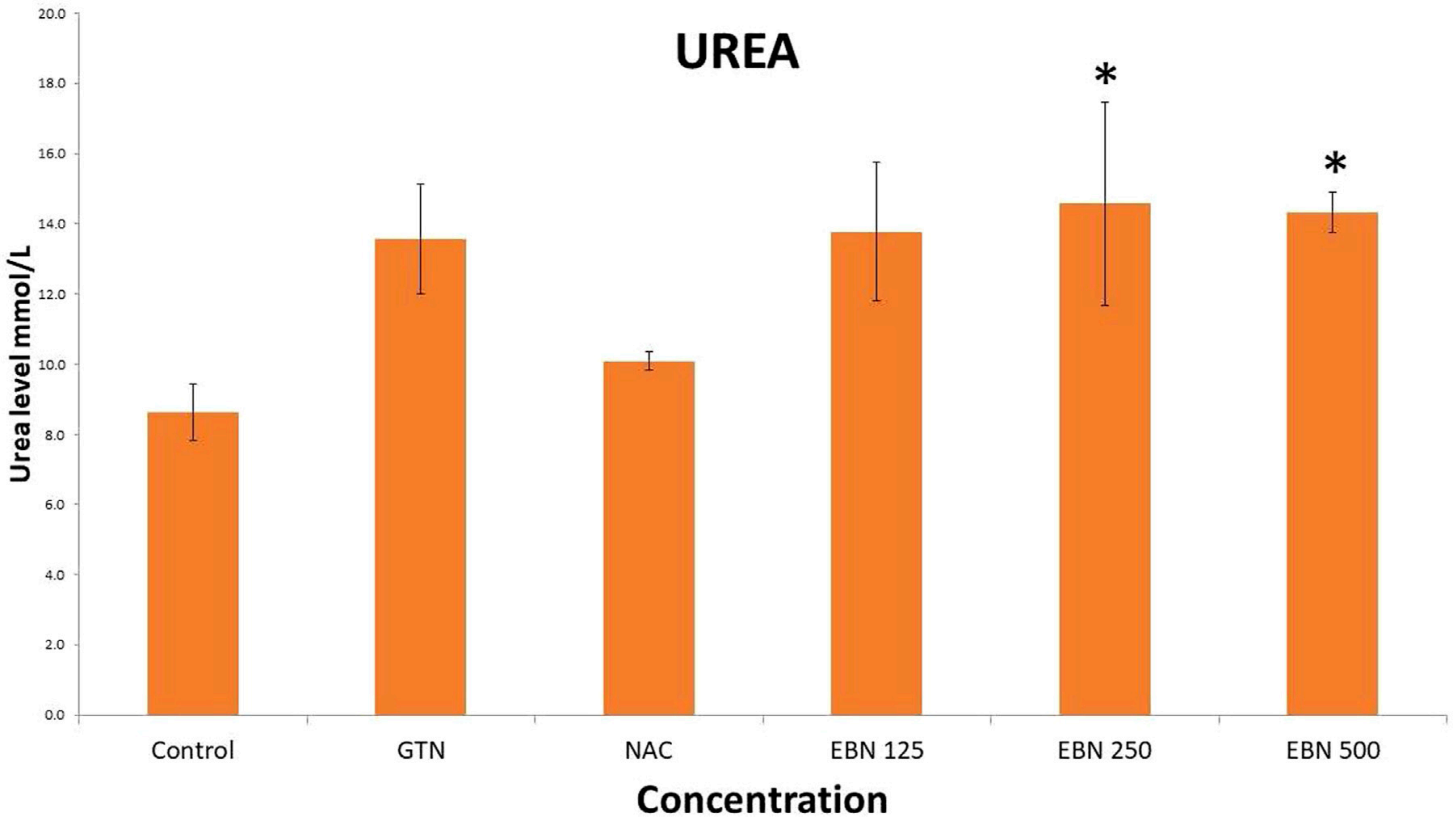
Changes of serum urea chnages vs different post-treatment group (*p < 0.05 compare to gentamicin group).

**FIGURE 13:**
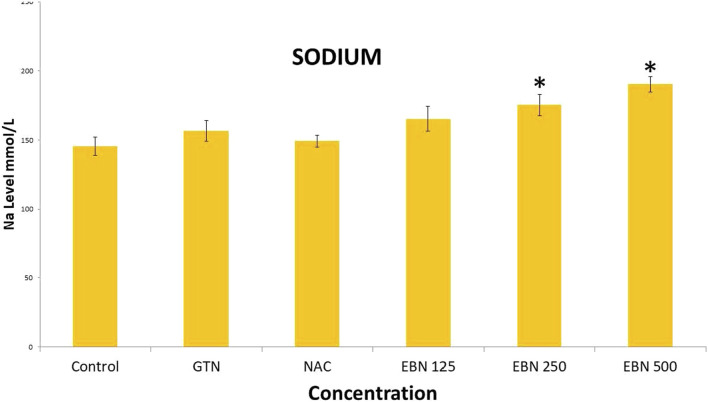
Changes of serum sodium vs different post-treatment group (*p < 0.05 compare to gentamicin group).

**FIGURE 14:**
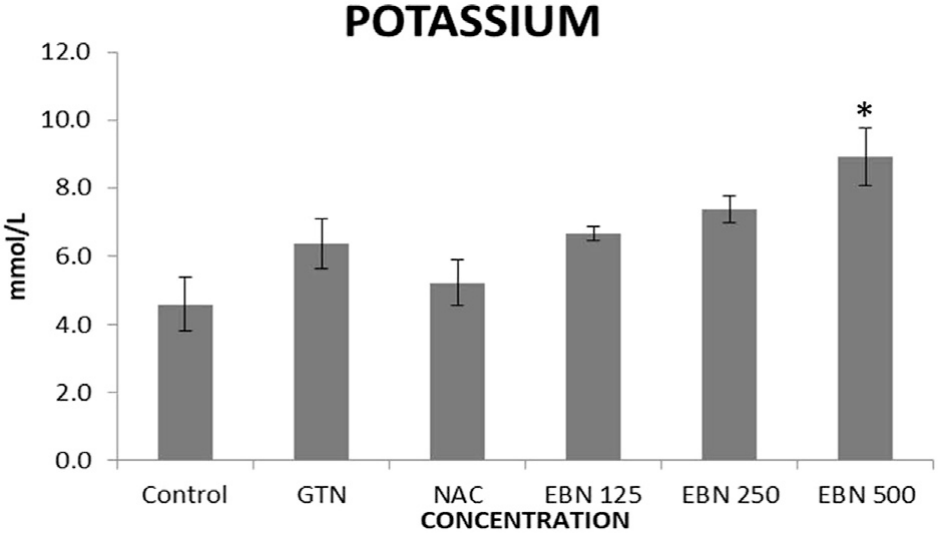
Changes of serum potassium vs different post-treatment group (*p < 0.05 compare to gentamicin group).

As for post treatment results, serum sodium, urea and creatinine were signficantly higher in the 250 and 500 mg/kg groups as compare to the GTN group. Serum urea levels did not show any significant changes. Serum potassium and chloride were significant increased in 500 mg/kg post treatment groups as compare to GTN (Figures 11–14).

### Chemical Analysis of EBN

By using HPLC, the sialic acid content of the EBN used in this study was 5.47%. In term of proximal analysis, the order of composition, from highest to the lowest is protein (55.5 ± 2.5%), carbohydrate (28.6 ± 6%), moisture (12.1 ± 1.6%), ash (2.8 ± 0.1%) and lipid (0.1%). In term of safety profile, there was acceptable limit of nitrate (35.9 ± 0.1 mg/kg) and nitrite level (11.4 ± 0.2 mg/kg). There were no trace of arsenic, mercury, lead or cadmium inside the EBN. AS for microbiology profile, there were no *E Coli, S. Aureus, Salmonella, Coliform* or mould detected. The full chemical analysis of the EBN used in our study can be obtained in our earlier works that had been published (Careena et al., 2018; Tan et al., 2020).

## Discussion

AKI is a sudden episode of loss of renal function that happens within a few hours or a few days. AKI increases the long-term risk of developing chronic kidney disease (CKD) or end-stage kidney disease (ESKD) (Hsu and Hsu, 2016). Moreover, the presence of AKI has been associated with an increase in short-term and long-term morbidity and mortality (Samuels et al., 2011). Aetiologies of AKI are usually divided into prerenal, renal and postrenal causes. Among hospitalised patients in medical wards, AKI usually comes from the prerenal aetiologies (volume depletion or low cardiac output) or acute tubular necrosis (ATN) (nephrotoxin, ischaemia or sepsis). Acute tubular necrosis (ATN) occurs when there is death of tubular epithelial cells that lined the renal tubules of the kidneys. It can only be accurately diagnosed via a renal biopsy.

One of the vital functions of the human kidney is the regulation of water and excretion of nitrogenous waste product generated by the body. Although the measurements of elevated blood urea and creatinine level traditionally serve as the surrogate marker of decreased renal function, they are not accurate per see. An acute rise in serum urea and creatinine can be attributed by hypercatabolic state, excessive protein intake, AKI, gastrointestinal bleeding or dehydration. In clinical practice and in any experimental animal model, a precise diagnosis of AKI will require the presence of ATN confirmed histologically *via* a renal biopsy rather than relying on traditional blood test or biomarkers (Kudose et al., 2018). In the clinical management of AKI, a combination of serum creatinine and histopathological analysis can accurately tell us about the state of AKI. The cytokines and other specific blood and urinary biomarkers (apart from serum creatinine) remain controversial, and most importantly, most of these markers compare their performance with regards to serum creatinine. In a systemic review carried out by our us, we have found that traditional markers like urea and creatinine still have significant roles in clinical management of AKI and CKD compare to emerging biomarkers (Bidin et al., 2019).

In our study, renal injury is induced by the usage of GTN, which causes direct nephrotoxicity. GTN is known to cause direct tubular damage through a combination of necrosis of tubular epithelial cells, predominantly in proximal segment and alteration of function of main cellular components involved in transport and conservation of water and solutes (Randjelovic et al., 2017). The degree and severity of ATN in the kidneys correlate with the recovery of renal function in the long term. The more severe the ATN, the higher chances that the patient will end up with chronic kidney disease.

Our renal histological examination demonstrated that pre-treatment with EBN protects the kidneys from the nephrotoxic effect of GTN, whereby the GTN mediated toxicity was either negated or severely attenuated. For those who had received post-treatment EBN, the renal tubules were similarly protected, albeit not as effective as pre-treatment arms, as modest ATN changes from GTN was observed.

While we were expected to see a corresponding improvement in renal profiles, serum urea and creatinine level were noted to be progressively increased in the pre and post EBN treatment rats despite improvement observed in the renal tissues examination. Of note, the serum urea measurements were disproportionately high as compared to the serum creatinine levels, a finding that suggests the presence of moderate to severe dehydration (define as the ratio of urea/creatinine >1: 20) (Tariq et al., 2009). To confirm the presence of dehydration, we have proceeded to analyse the serum osmolarities of the post-treated samples which showed a trend of increasing osmolarity from 315.9 ± 23.6 mOsm/kg from the group that received 125 mg EBN to the highest of 424 ± 13.4 mOsm/kg for the group that received 500 mg EBN, therefore confirming that the elevated serum urea and contribution were likely to be attributed by dehydration.

As recovery of ATN begins as early as hours after the insults, we designed this study hoping the be able to observe the trend of improving creatinine after 7 days. Unfortunately, we did not see such a trend from the blood test. Animals that have ATN are known to lose the water concentrating ability with polyuria as a norm. Full recovery or the kidney tissues from ATN is expected in 14–60 days after the initial insult. Nancy et al. advocated a full renal recovery will require an average of 42 days (Gary et al., 1976). Hence in retrospective, our protocol that used serum renal profiles as a measurement of kidney function will not be a suitable method to assess renal function in such a short duration of an experiment like ours. We strongly proposed that future similar trials should have a minimum of 14 days post intervention to have a better assessment of renal recovery.

Although EBN is widely believed to have protective effect towards the kidney for many centuries, ours is the first that successfully documented the reno-protective effect of EBN. This reno-protective effect is believed by our research team to be effected through the presence of epidermal growth factor which repair the damaged tissues, maintenance of renal tissue integrity via the sialic acid and lastly, down regulation of the pro-inflammatory pathway.

We postulated that the reno-protective effect of EBN could be attributed to the presence of sialic acid and epidermal growth factors. As our group has reported previously, our EBN has a good amount of sialic acid (Careena et al., 2018). Sialic acid is essential for maintaining renal function. Appropriate sialylation of the glomerular filter is vital for proper maturation and maintenance of the visceral part of the kidney filtration apparatus. Sialylation, through oral sialic acid supplementation therapy, has gained new interest as a potential new therapeutic strategy to cure or delay glomerulopathies such as focal segmental glomerular sclerosis (Niculovic et al., 2019). Apart from preserving the glomerular cells, sialic acid also plays a vital role in maintaining the integrity of renal tubular cells (Sgambati et al., 2011).

Reside inside kidney tissues is a variety of immune cells such as dentritic cells, macrophages, regulatory T cells, which play an important role in the maintenance of tissue homeostasis. Once activated by external trigger such as LPS, these cells produce proinflammatory cytokines that can initiate and propagate the kidney disease which can culminate in irreversible renal scarring (Andrade-Oliveira et al., 2019). EBN can inhibit the generation of proinflammatory cytokines (TNF-α, IL-1*β*, and IL-6) and oxidative markers. Pre-treatment of sialic acid significantly lessened the LPS-induced detrimental effects on the renal haemodynamics, renal reactive oxygen species production and the LPS-activated TLR4/gp91/Caspase3 mediated apoptosis signalling (Hsu et al., 2016).

EBNs are also believed to contain epidermal growth factors (EGFs) (Tan et al., 2020). EGFs are small mitogenic proteins involved in several mechanisms, such as healthy cell growth and differentiation. Recently EGFs have been found to regulate sodium transport and development of hypertension. EGF receptor activation has been shown to ameliorate renal damage sustained in experimental AKI through the repairing process that involves the regeneration of the renal tubular cells (Melenhorst et al., 2008; Rayego-Mateos et al., 2018).

The limitation of our study is the short duration of the animal study and the failure to keep the animal’s adequate hydration during the experiment. However, we have an excellent and comprehensive histological assessment of the renal tubules, which has provided a comprehensive and real time information about renal tubular structure changes during the experiment. Despite the shortcomings, in this pilot trial, we have demonstrated that pre and post treatment with EBN protects the gentamicin treated kidneys from developing ATN. We hope that this preliminary finding can lead to further in depth study of the possible mechanisms of reno-protective properties of EBN.

## Conclusion

This study revealed that pre-treatment of EBN is a more effective means of preventing GTN induced ATN as compared to post-treatment with EBN. We believe that the renal protective effect could be due to the presence of sialic acid, which is needed to maintain a proper kidney function. Similarly, EGF could have assisted in the regeneration of the renal tubules post-ATN. Further studies are needed to assess whether external supplementation of EBNs can render reno-protection from other models of AKI or chronic kidney disease.

## Sample storage

Samples are stored at Pharmacotherapeutics Laboratory, Faculty of Medicine and Health Sciences, Universiti Putra Malaysia for posterity purpose.; Ethics approval.

## Data Availability

The original contributions presented in the study are included in the article/Supplementary Material, further inquiries can be directed to the corresponding author.

## Abbreviations

AKI, acute kidney injury; ATN, acute tubular necrosis; CKD, chronic kidney disease; EBN, edible bird’s nest; ESRF, end stage renal failure; NAC, N-acetylcysteine; GTN, gentamicin
